# Nutritional Disorders and Metabolic Adaptations in Dromedary Camels: Insights into Foregut Fermentation and Mineral Balance

**DOI:** 10.3390/ani16040689

**Published:** 2026-02-23

**Authors:** Muhammad Mahboob Ali Hamid, Mohamed Tharwat, Tarek A. Ebeid, Fahad A. Alshanbari

**Affiliations:** 1 Institute of Animal and Dairy Sciences, Faculty of Animal Husbandry, University of Agriculture, Faisalabad 38040, Pakistan; dr.mmahboob@uaf.edu.pk; 2 Department of Clinical Sciences, College of Veterinary Medicine, Qassim University, Buraidah 51452, Saudi Arabia; 3 Department of Animal and Poultry Production, College of Agriculture and Food, Qassim University, Buraidah 51452, Saudi Arabia; t.ebeid@qu.edu.sa; 4 Department of Medical Biosciences, College of Veterinary Medicine, Qassim University, Buraidah 51452, Saudi Arabia

**Keywords:** dromedary camel, foregut fermentation, metabolic disorders, nutrition, oxidative stress

## Abstract

Dromedary camels possess unique anatomical, physiological, and metabolic adaptations that enable survival in arid environments; however, these same adaptations make them highly sensitive to nutritional imbalance under modern feeding conditions. This review synthesizes current knowledge on nutritional pathologies and metabolic disorders in camels, emphasizing the links between diet composition, foregut fermentation, mineral status, and systemic health. Imbalances in energy and carbohydrates predispose camels to subacute and acute acidosis, negative energy balance, and ketosis-like syndromes, particularly when rapidly fermentable feeds are introduced without adequate fiber or water. Protein and nitrogen disorders, including ammonia toxicity and impaired urea recycling, arise from mismatches between degradable protein, fermentable energy, hydration, and mineral availability. Widespread deficiencies of phosphorus, copper, cobalt, zinc, selenium, and vitamins A and E remain major constraints, leading to pica, poor microbial fermentation, oxidative stress, immunosuppression, reproductive failure, and skeletal disorders. Nutritional disturbances frequently extend beyond the gastrointestinal tract, forming a gut–liver–kidney metabolic axis characterized by hepatic dysfunction, renal compromise, and systemic oxidative stress. The review also addresses gastrointestinal impaction, foreign-body ingestion, toxic plant consumption, and feeding on human food waste as emerging nutritional challenges, particularly in peri-urban systems. Advances in diagnostic ultrasonography, feed evaluation techniques, probiotics, mineral–vitamin supplementation, and omics-based approaches are discussed as tools for improving early diagnosis and precision nutrition. Despite growing research interest, the lack of camel-specific feeding standards and reliance on cattle-based recommendations remain critical gaps. This review highlights the need for species-specific nutrient requirement models, sustainable rangeland management, and integrative research to support the health, resilience, and productivity of camels under changing environmental and production systems.

## Simple Summary

Dromedary camels are well adapted to harsh desert environments, but modern feeding practices, climate stress, and poor-quality forages have increased the risk of nutritional and metabolic disorders. This review explains how imbalances in energy, protein, minerals, and vitamins affect camel health and productivity. Sudden dietary changes, excessive concentrates, limited water, and mineral-deficient rangelands commonly lead to problems such as acidosis, impaction, pica, oxidative stress, reduced milk production, and reproductive failure. Unlike cattle, camels have slower fermentation, limited buffering capacity, and unique nitrogen recycling, making them particularly sensitive to inappropriate feeding strategies. Nutritional disturbances often extend beyond the digestive system and affect the liver and kidneys. The review also discusses the role of toxic plants, garbage feeding, and environmental stressors in worsening nutritional disease. Recent advances in feed evaluation, diagnostic imaging, and molecular tools offer promising ways to detect nutritional stress early. Overall, this review highlights the need for camel-specific feeding guidelines, balanced mineral supplementation, and sustainable feeding practices to improve camel welfare and productivity.

## 1. Introduction

Dromedary camels (*Camelus dromedarius*) are very important in food security, livelihoods, and ecological sustainability in arid and semi-arid areas of Africa, the Middle East, and South Asia [1,2]. The unusual anatomical, physiological, and metabolic changes that enable them to survive and stay productive in conditions of extreme heat, extended water deprivation, and low-quality forage are the basic factors that essentially separate them from the true ruminants [3,4,5]. Consequently, camels have been introduced more and more in pastoral, semi-intensive, and intensive production systems, especially with milk, meat, transportation, and racing [4,5].

Although the economic relevance of camels has continued to rise, the nutritional requirements of camels have not yet been properly defined and mentioned, often being generalized based on cattle- or small-ruminant-based feeding regimes, which do not consider the digestive physiology and metabolic control of camels [4,6,7,8]. Camels have a special system of the foregut (C1–C3 compartments), long retention time of the digesta, high nitrogen recycling, and developed mechanisms of water and electrolyte conservation [7,9]. Although these adaptations grant outstanding resistance to defiant environmental factors, they also precondition the occurrence of chronic and insidious nutritional and metabolic diseases in camels when they are subjected to a wrong feeding plan, high-speed dietary change, or intensive custodial conduct [10,11,12].

During the past few decades, the shift in the camel production systems, including the increased use of concentrates, irrigated fodders, and limited grazing coupled with peri-urban grazing on human waste, has been linked with the increase in nutrition-related disorders [13,14,15]. They are foregut dysmotility, impaction, subacute acidosis, mineral and vitamin deficiencies, oxidative stress, reproductive failure, and renal-hepatic dysfunction [16,17,18,19,20]. However, in the case of camels, unlike cattle, they show retarded or abnormal clinical presentation because of their conservative fermentation profiles, stable glucose homeostasis, and an efficient lipid mobilization mechanism that makes them especially difficult to diagnose and prevent early onset [21,22,23,24,25].

Despite the fact that there is a significant amount of literature on camel digestive physiology and metabolic adaptations, it is still applicable across fields, with little to no integration of fundamental physiology, feeding systems, nutritional needs, and clinical pathology [24,26,27,28,29]. In addition, there are limited camel-specific nutrient requirement models and no universally agreed feeding standard for camels, especially in conditions of intensive or semi-intensive feeding [21,30,31,32,33]. The absence of synthesis will lead to the further use of cattle-based recommendations, which can increase metabolic stress and undermine animal health and productivity [29,33,34].

Thus, the purpose of this review is to critically synthesize the existing information related to nutritional disorders and nutritional metabolism in dromedary camels and pay specific attention to the relationships between foregut fermentation, nutrient metabolism, mineral-vitamin balance, and systemic health [6,7,35,36,37]. In this review, the evidence provided by camel-specific research, as well as well-chosen comparative studies, is combined to explain the mechanisms of nutrition-related diseases and clinical implications. This review synthesizes physiological and clinical evidence to identify consistent patterns, contradictions, and knowledge gaps in camel nutrition research.

## 2. Materials and Methods

### 2.1. Review Protocol and Reporting Standards

The review and reporting were carried out and presented in line with the Preferred Reporting Items of Systematic Reviews and Meta-Analyses (PRISMA) 2020 statement. An a priori developed structured review protocol was used to identify, screen, evaluate the eligibility of the studies, and synthesize them.

### 2.2. Eligibility Criteria

The criteria to include the studies were: (i) the studies should have involved camel dromedaries (Camelus dromedarius) or camelids with direct physiological significance; (ii) the studies should have included nutrition, metabolism, mineral balance, feeding systems, or nutrition-related disorders; and (iii) the studies should have been original research articles, clinical studies, or expert reviews in peer-reviewed journals. Exclusion criteria were non-camelid studies with no translational relevance, anecdotal case reports without data in their analysis, and studies that did not deal with nutritional or metabolic outcomes.

### 2.3. Search Strategy and Information Sources

In PubMed, Scopus, and Web of Science Core Collection, systematic searches were done of articles published between January 1990 and June 2025. The reference lists of key reviews and textbooks were also searched manually. The search technique was a combination of both controlled vocabulary and free-text words: Camelus dromedarius, camel nutrition, foregut fermentation, mineral deficiency, metabolic disorders, oxidative stress, acidosis, urea recycling, etc.

### 2.4. Study Selection Process

The titles and abstracts were then screened by two reviewers after the duplicates were eliminated. Inclusion criteria were used to evaluate the full texts of potentially eligible articles. Dissenting issues were addressed through arguing and agreement.

### 2.5. Data Mining and Data Evaluation

Standardized forms, such as the design of the study, population characteristics, nutrition exposures, metabolic outcomes, and key findings, yielded relevant data that were extracted. Since the study designs were heterogeneous, there was no formal meta-analysis done. The quality of the study and internal validity were evaluated based on modified STROBE- and ARRIVE-based criteria, where the study was applicable.

### 2.6. Data Synthesis

A narrative synthesis format was utilized, which was organized based on thematic areas such as physiology of the foregut, energy and protein metabolism, mineral and vitamin homeostasis, oxidative stress, feeding systems, and clinical pathologies. The comparison of evidence across the studies was done to find convergent mechanisms, inconsistencies, and gaps in research.

### 2.7. PRISMA Flow of Study Selection

**Figure 1:**
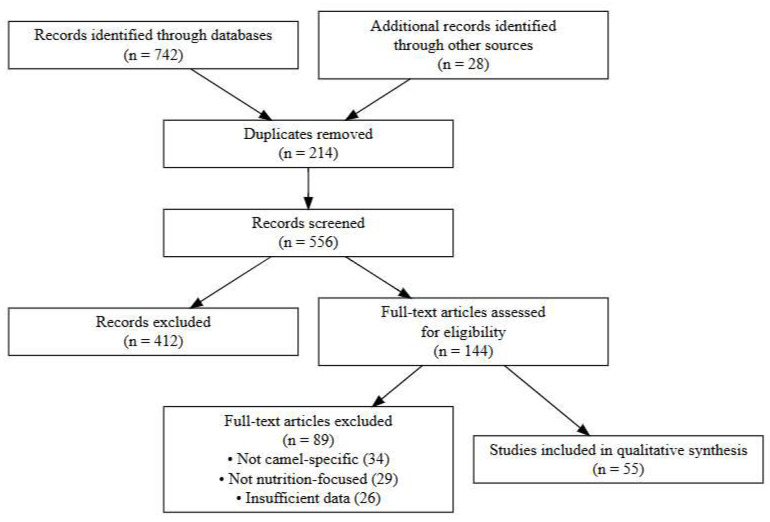
PRISMA 2020 flow diagram of literature identification, screening, and inclusion. The diagram illustrates the selection process for studies included in this review, including database searching (PubMed, Scopus, and Web of Science), duplicate removal, title and abstract screening, full-text assessment, and final inclusion in the qualitative synthesis. The review followed PRISMA 2020 reporting principles to ensure transparency of study selection.

The search of the literature revealed 770 records (742 database records and 28 in manual search). Upon eliminating 214 cases of duplication, 556 cases were then filtered by the title and abstract of the records, and 412 cases were eliminated. The 144 articles were evaluated using their full text, and 89 of these articles were excluded because they were not specific to camels, did not offer the necessary nutritional relevance, or had methodological constraints. The final qualitative synthesis of 55 studies was found (Figure 1).

## 3. Essentials of Camel Nutrition

### 3.1. Anatomy of the Camel Stomach (C1–C3 Compartments)

The functional integration of these compartments allows prolonged digesta retention and efficient fermentation under arid conditions [38,39,40]. The compartment C1 is the largest one and occupies almost 80 percent of the total stomach mass, which is functionally parallel to the rumen of the bovines [41,42]. Quantitative anatomical studies indicate that C1 accounts for approximately 75–80% of total gastric volume in adult dromedary camels, compared with 60–65% rumen volume in cattle, explaining the prolonged digesta retention and fermentation stability observed under low-quality forage conditions [40,43,44]. It takes up the majority of the left abdominal cavity and is primarily involved in microbial fermentation of structural carbohydrates. Sacculated regions within C1 facilitate prolonged fluid retention, supporting fermentation stability during extended periods of water deprivation [38]. C1 has a stratified squamous epithelium that offers mechanical defense against coarse and lignified feeds and the extensive papillary network, which enhances the surface area, and absorbs volatile fatty acid (VFA). The sacculated architecture has been recently affirmed to increase the buffering and selectivity of feedstuff retention by anatomical characterization of ruminants, which improves the digestion of roughage in the camel compared to cattle and small ruminants [39].

C2 contributes to digesta sorting, regurgitation, and buffering, supporting fermentation continuity under fluctuating dietary conditions [45]. The distal region of C3 exhibits glandular secretory activity comparable to the abomasum, enabling effective enzymatic digestion and mineral solubilization [4,38,40]. Another anatomical peculiarity of the camel stomachs is the widespread vascularization and muscular control, allowing the storage and re-use of the fluid. These gastric water cells are able to hold over 20–30 L of water and maintain luminal moistness when they are not drinking water [38,40]. Experimental dehydration–rehydration studies demonstrate that camels can safely ingest 80–100 L of water within minutes without inducing osmotic shock or foregut dysfunction, a capacity far exceeding that of cattle [39,43,44].

Compared with true ruminants, camelids exhibit a simplified yet functionally integrated foregut system that prioritizes prolonged digesta retention, water conservation, and fermentation stability under resource-limited conditions. These differences underpin the camel’s nutritional resilience but also necessitate species-specific feeding strategies [1,4,14,46,47,48]. The actual ruminants do not have such special reservoirs and are rapidly killed by water deficiency. One of the significant evolutionary benefits that allows continuous fermentation despite external deprivation of water is therefore the existence of water cells.

### 3.2. Physiology of Fermentation and Fluid Dynamics in the Camel Stomach

The interlocking of the activities of C1 and C2 and the proximity of C3 form a synergized fermentation chamber that is designed to conserve selectively, lignify microbial degradation of vegetation efficiently, and conserve water; see Table 1. Mean C1 pH values in healthy camels are typically maintained between 6.2 and 6.8, even during feed restriction or dehydration, whereas cattle commonly show pH declines below 6.0 under similar dietary challenges. Retention time of solid particles in camels has been reported to exceed 70–90 h, compared with approximately 40–60 h in cattle [46,49,50]. In dromedary camels fed mixed forages, ruminal ammonia (NH_3_-N) concentrations and pH vary significantly with diet composition, with maximum NH_3_-N observed 2–4 h post-feeding and pH changes reflecting diet type. Rumen VFA concentrations were significantly influenced by C3 vs. C4 forage composition, with C3 diets yielding higher total VFA production (*p* < 0.05), underscoring diet effects on fermentative metabolism [51,52].

The fermentation in the camel stomach takes place mainly in C1 and C2, where the symbiotic micro-organisms metabolize structural carbohydrates to VFAs, microbial protein, gases, and heat. The mucosal glands of C1 and C2 secrete bicarbonate-rich fluid that neutralizes fermentation acids, normalizes the pH, and promotes the functionality of the microbes. The turnover of fluid in the foregut is lower in camels than in cattle and leads to the maintenance of microbial retention and enhancement of fibrous forage digestion.

Camels are highly adaptive to changes in plasma osmolality, and this can be seen in the forestomach sections of their bodies. They are able to endure blood osmolality increases, which would severely dehydrate other species, and can freely uptake highly concentrated VFAs and water in saline forage; see Table 1. The fluid in C1 and C2 may become hyperosmotic without affecting fermentation, partly because of halotolerant microbes and adaptive epithelial transport systems [53,54,55].

These physiological and metabolic peculiarities explain the significance of camel-related nutritional interventions, the early detection of chronic digestive disorders, and the need to interpret the biochemical and ultrasonographic data in the clinic carefully. Tharwat [53,54,56] notes that clinicians should not use cattle-based diagnostic and feeding principles for camels since these methods will not consider the unique resilience of the metabolism of the species and, at the same time, latent weaknesses. Practical interpretation of these variations is crucial in the development of proper diets, prevention of chronic gastrointestinal disease, and enhanced metabolic health in traditional as well as intensive camel production systems; see Table 1.

**Table 1:** Structural and functional characteristics of the camel foregut compartments (C1–C3).

Domain	Camel-Specific Mechanism	Underlying Physiological Basis	Nutritional Consequence	Clinical Implication	References
Water and Osmotic Balance	Exceptional tolerance to dehydration and hyperosmolar plasma	Elastic erythrocytes; strong renal concentrating ability	Maintains C1 fermentation during dehydration	Higher risk of impaction during drought	[26,27,57,58,59]
Lipid Mobilization	Efficient adipose utilization with limited ketosis	Controlled ketogenesis; strong β-oxidation	Maintains energy supply during feed shortage	Chronic weight loss rather than acute ketosis	[58,60]
Carbohydrate Metabolism	Stable glucose turnover during fasting	Gluconeogenesis dominance; controlled VFA release	Reduced hypoglycemic episodes	Delayed anorexia signs	[42,59]
Nitrogen Recycling	Highly efficient urea recycling to C1	Upregulated urea transporters and microbial urease	Better survival on low-protein desert diets	NPN misuse → ammonia toxicity	[9,61,62,63,64]
Fermentation Physiology	Slow conservative VFA production	High fibrolytic bacterial activity	Lower risk of acute acidosis	Chronic dyspepsia more common	[63,65]
Mineral Homeostasis	Sensitive to P, Cu, Zn, and Se deficiencies	Desert forage mineral scarcity	Frequent field deficiencies	Pica, infertility, oxidative stress	[12,16,17,20, 66,67,68]
Heat Tolerance	Low heat load, high thermal resistance	Reduced sweating, altered hypothalamic set-points	Lower maintenance energy	Hidden dehydration → GIT dysmotility	[5,69,70,71]

This table summarizes the anatomical organization, relative volume, epithelial features, and primary physiological roles of the camel stomach compartments, highlighting differences from true ruminants relevant to digestive efficiency and nutrient retention. Abbreviations: C1, first stomach compartment; C2, second stomach compartment; C3, third stomach compartment.

Camel stomach anatomy and physiology have direct and significant implications on feeding and nutrient use and clinical presentation of nutritional diseases in dromedaries. Since C1, C2, and C3 combine the actions of rumen-reticulum and omasum-abomasum, the nutritional intervention acting in cattle or small ruminants cannot be transferred to camels linearly without endangering metabolism and decreasing productivity [40,45,72]. Nutritionally, this enables camels to use low-moisture, salt-tolerant desert forages without having to add water to them [55].

## 4. Metabolic and Digestive Adaptations of Dromedary Camels to Arid Environments

### 4.1. Metabolism in Camel

#### 4.1.1. Energy and Carbohydrate Metabolism in Dromedary Camels

Energy metabolism in dromedary camels is shaped by selective pressure toward metabolic stability rather than maximal productivity, a distinction that has direct implications for nutritional management and disease expression under arid conditions. These adaptations allow camels to tolerate prolonged nutritional and environmental stress while masking early clinical signs of negative energy balance, complicating field diagnosis compared with cattle [69,73].

#### 4.1.2. Volatile Fatty Acid Synthesis and Metabolism

In camels, VFA production is characterized by slow, conservative fermentation kinetics and a consistently acetate-dominant profile. This pattern reduces susceptibility to acute ruminal acidosis but predisposes animals to chronic dyspepsia and subclinical energy deficits when diets are improperly intensified.

#### 4.1.3. Homeostasis of Glucose and Gluconeogenesis

The primary precursor of hepatic gluconeogenesis is propionate, which is produced by microbial fermentation. However, during periods of reduced feed supply, camels also mobilize glycerol from fat stores and certain amino acids [74,75]. The secretion of insulin is lower in the basal levels in the camel pancreas compared to the cattle, and the peripheral tissues are also less sensitive to insulin; this is an adaptation that helps to pay more glucose to essential tissues like the brain and the mammary glands. Even in the case of long-term fasting, camels do not experience the extreme hypoglycemia common to underfed cattle, and this again illustrates their metabolic plasticity [76].

#### 4.1.4. Heat Stress and Energy Partitioning

Camels also have heat-adaptive metabolic mechanisms that reduce the amount of energy that is lost during hot seasons. Camels do not exhibit a drastic reduction in the feed and fermentation under heat alleviation, as cattle do. Their reduced metabolic heat increment decreases the thermogenic cost of digestion, which enables them to consume the fibrous feeds without overheating [69,77]. During extreme heat, the metabolic rate decreases, the turnover of glucose is enhanced, mechanisms of saving water minimize salivary loss, and the motility of the foregut is slowed in order to maintain luminal moisture. The effects of these shifts can save energy consumption and keep the microbial activity alive, so camels can sustain milk production and body state despite the stress that causes cattle to deteriorate [55,60,63].

#### 4.1.5. Increased Focus and Glucose Intolerance

In spite of the fact that camels are highly adaptive to low-quality forages, they do not react to a large degree to rapidly fermentable carbohydrates. Excessive intake of concentrate can lead to subclinical acidosis, C1 dysmotility, gas accumulation, feed refusal, and chronic indigestion. In more severe cases, it may also result in C3 ulceration [6,78,79,80]. Since the camel C1–C2 system has more lagging buffering and a conservative motility pattern than cattle, the sudden rises in starch interfere with fermentation deeper. Consequently, concentrates are to be added gradually, and roughage forms the central part of camel diets even in intensive dairy production.

### 4.2. Metabolism of Protein and Nitrogen in Dromedary Camels

The determination of the maintenance energy and protein needs of adult dromedary camels has been conducted in controlled feeding and nitrogen balance trials. Based on allometric scaling, the maintenance metabolizable energy requirement in adult camels (weighing 292–715 kg) has been estimated at approximately 104 kcal ME/kg^0.75/day, while the maintenance digestible nitrogen (DN) requirement—accounting for protein–energy interactions—has been calculated at 368 mg DN/kg^0.75/day (taking protein–energy interactions into account) in adult camels weighing from 292 to 715 kg. The figures observed are lower than the recommended allowances for cattle and buffalo, yet they are similar to those for sheep when adjusted for metabolic body size, which demonstrates the effective capacity of camels to conserve nutrients [81].

#### 4.2.1. Conservation of Nitrogen and Recycling of Urea

The ability of camels to be intensive urea recyclers was the first distinction between camels and true ruminants and was first reported by early physiologists like [41] and subsequently validated by [42]. Loss of dietary protein—a frequent situation in desert habitats—causes a catastrophic augmentation of C1 and C2 plasma urea transfer in camels. Urea is then broken down by microbial ureases to ammonia that is utilized by the microbial protein. This recycled nitrogen promotes microbial growth despite the presence of absolutely low levels of dietary protein intake and therefore permits camels to sustain fermentation efficiency and nitrogen equilibrium during drought phases, migration, or substandard pasture [81].

#### 4.2.2. Foregut and Protein Synthesis of Microbes

A large part of the amino acids that are absorbed by camels is microbial protein. The C1 lengthened retention and reduced velocity are conducive to microbial growth, mostly fibrolytic and cellulolytic micro-organisms that develop on lignified shrub species, halophytic plants, and coarse roughage. Recycled urea is converted to microbial nitrogen by giving the best environment due to the stability of fermentation pH and buffering action of C1 water cells [27,61,62,82]. Due to this fact, camels are able to have a constant rumen ammonia level compared to cattle fed inconsistent protein sources [58,60,64].

#### 4.2.3. Amino Acid Professional Requirements and Metabolism

In the experimental trials, dromedary camel nitrogen retention, which was found to be greatly affected by the dietary quality and its intake in the range of −85 to +556 mg DN/day/kg^0.75, clearly depicts the nitrogen balance variations, thus resembling the metabolic changes in low-quality forages and water shortages. However, dairy camels, racing camels, and growing young animals during high production levels should be fed a balanced diet of amino acids to enhance lactation, growth, and tissue regeneration. Lack of the key amino acids, in particular lysine and methionine, can affect the ability to produce proteins in milk, immune activity, and reproductive performance. In contrast to cattle, where the protein-energy imbalances easily give way to ketosis, camels rather show hump fat mobilization and progressive depletion of protein stores, and these may not be accompanied by metabolic catastrophe [9,10,83].

#### 4.2.4. Protein Metabolism in Stresses of Heat and Water Deficit

Environmental stress is a strong factor in the protein metabolism of camels. When dehydrated, camels reduce their excretion of urea significantly and recycle more urea to sustain the intake of nitrogen to the microbes despite reduced feed intake. Heat stress decreases C1 motility and salivary flow and retards the entry of protein into the lower digestive tract. However, camels do not experience the rapid rise in the endogenous catabolism of nitrogen as observed in cattle when thermoregulatory demands are combined with a high-water conservation efficiency [63].

In extended water dehydration, camels develop reduced urinary nitrogen secretion, a saving of plasma amino acids, less degradation of body proteins, increased use of fat oxidation, and reduced use of proteolysis. These processes enable the camels to escape the tough catabolism of muscles and sustain physiological functioning [57].

### 4.3. Lipid Metabolism and Hump Fat Mobilization

Another advantage that camels show is their ability to mobilize adipose tissue when there is a deficit of feed, which allows the animal to maintain energy levels without developing severe ketosis, which is typical of high-producing cattle. This means that with their special lipid metabolism, they can effectively oxidize fatty acids during β-oxidation and regulate ketone body formation, thus avoiding the acute ketosis syndromes seen in dairy cows in the negative energy balance [53].

Among the carcass adaptations of camels is a massive mobilization of lipids from hump adipose tissue. During periods of energy inadequacy, camels store energy gradually and effectively without progressing to clinical ketosis, a condition that is common in high-producing dairy cows that are exposed to negative energy balance. The cause of this resistance to ketosis is due to a reduction in the rate of ketone bodies, increased peripheral ketone consumption, gradual, moderate fat release as opposed to sudden lipid release, and regulated β-oxidation of the hepatocyte [64,65]. The acetate pre-eminence of the camel VFA profiles indicates the existence of long-term lipogenesis in nutritional abundance periods, thus rendering the hump an effective long-term energy store. The mobilization of hump fat is helpful in periods when the feed is inadequate to sustain energy, therefore preventing an extreme burst in ketone concentration [63]. The acetate type is found, which promotes lipid metabolism and endurance of prolonged walking that is typical of nomadic movement [73,84,85].

### 4.4. Fiber Consumption and Microbial Ecology in Dromedary Camels

The high sacculations of C1 give more space to be colonized by microbes and absorb VFA, and the slow motility pattern lengthens the retention time, which then provides ample time to ferment [40,70]. The camels have higher particle retention times as compared to cattle, and this enhances the digestion of cell walls, particularly lignocellulosic forage common in desert ecosystems. Extended contact of plant material with microbial enzymes enables camels to gain as much energy as possible out of cellulose, hemicellulose, silica, tannins, and secondary compounds containing substrates. Foregut motility is also adequate to sustain fiber degradation even in dehydration or heat stress conditions, and this illustrates the strength of the camel digestive system [4,23].

The characteristics produce a fermentation system that is ideal for the transformation of not-good-yet-forage to VFAs, microbial protein, and energy. Also, camel microbiota is less sensitive to pH variations and osmolality fluctuations in comparison to cattle, which makes it less likely to collapse fermentation in the event of water limitation or feed seasonal unavailability [11,33,86].

In the process of digesting fibers, camels depend on the restoration of nitrogen in facilitating microbial synthesis of proteins. The re-use of urea as C1 and C2 guarantees the constant supply of nitrogen despite a very low content of crude protein in the forage, which is a common circumstance in desert pastures. This internal buffer of nitrogen enables the fibrolytic bacteria to remain active without such sharp falls in rumen ammonia levels familiar with cattle fed low-protein rations [4,87,88,89].

## 5. Feeding Systems and Nutritional Management of Dromedary Camels

### 5.1. Extensive (Pastoral) Feeding Systems

Transhumant and nomadic camel herds usually consume a great diversity of desert and semi-desert vegetation, such as grasses, shrubs, halophytes, thorny species of Acacia, and drought-tolerant forbs [29]. Pastoral systems are able to operate even when there is a severe shortage of forage due to their capacity to eat thorny vegetation without injuring their mouths and mobilize hump fat. Seasonal variation in the quality of forage is a characteristic of pastoral systems. Camels typically become fatter, and a buildup of hump stores occurs during the rainy season as a result of a higher supply of green vegetation, protein, and carotenoids. Conversely, nutrient supply drastically decreases during the dry season. The amount of protein in desert forages can drop to less than maintenance, and the amount of energy in them decreases with plant age and fibrousness. In this case, the high level of nitrogen recycling of camels [9] is vital to maintain microbial activity and avoid severe protein deficiency.

Depending on the temperature (10–50 km/day), camels can graze over a distance of 10–50 km and can last several days and weeks without drinking. Fermentation is also maintained under dehydration by their stomach sacculation, the physiological mechanism of water retention, and their tolerance to high plasma osmolarity [12,90]. This provides camels with a strong survival edge over cattle that develop fast rumen dysfunction in the absence of daily water consumption. The camels are also exposed to high levels of variability in mineral intake through pastoral systems. Numerous desert soils lack phosphorus, cobalt, and copper, and halophytic plants can provide surplus sodium and sulfur.

### 5.2. Semi-Intensive and Intensive Dairy Camel Systems

Camels that are not well-balanced with supplementation, especially with the composition of proteins, fiber, and minerals, are likely to develop such disorders as chronic C1 dysmotility, mild acidosis, urolithiasis, or mineral-related infertility [11,91]. The diets typically comprise alfalfa hay, silage, concentrates (barley, oats, corn), protein meals (soybean, cottonseed), and high milk yield vitamin-mineral mixes. These systems facilitate the year-round production and the regulation of breeding, as well as create new metabolic issues that camels have not previously faced in their desert grazing environment [12,87].

High-concentrate feeding is one of the most significant risks of intensive systems, and it may be more than the naturally available tolerance of the camel to rapidly fermentable carbohydrates. Camels are more prone to: owing to slow relative foregut motility, sparse buffering secretions, and economical fermentation; they have conservative fermentation patterns. That leads to subacute foregut acidosis, gas accumulation, chronic dyspepsia, C1 atony, and C3 ulcers. The danger is enhanced when the dietary fiber is inadequate or the transition of the type of ration is too fast [34,70,92]. Therefore, the incorporation of concentrate should be done slowly, and fiber diets should be the main constituent of camel diets even in intensive management. The improvement of recycling of nitrogen enables camels to sustain microbial protein production when feeding on low-protein diets, lowering crude protein levels of camels compared to those of cattle [9]. Nevertheless, the balanced feeding of dairy camels with low protein does not exclude the need of balanced amino acid supply for high-producing dairy camels because milk production increases the nitrogen requirements.

### 5.3. Seasonal Fluctuations and Drought Impacts on Camel Nutrition

The nutritional status, health, and productivity of dromedary camels in arid and semi-arid zones are significantly affected by seasonal variations. Desert ecosystems undergo extreme contrasts between wet and dry seasons, leading to substantial changes in forage quantity, quality, and botanical composition [29]. Increased forage moisture also boosts ruminal fermentation efficiency, volatile fatty acid production, and foregut motility, contributing to peak milk production [93,94].

Conversely, the dry season is marked by severe feed shortages, with pastures dominated by lignified shrubs, thorny Acacia species, dry grasses, and salty halophytes, which are low in crude protein (often below 4–6%), phosphorus, and trace minerals [61,95]. Camels rely on metabolic adaptations such as enhanced urea recycling, slower digesta passage, and mobilization of hump fat reserves to maintain energy homeostasis [27,82]. Nevertheless, prolonged dry periods lead to deteriorated body condition, decreased milk yield, reduced fertility, compromised immunity, and increased prevalence of parasitic and infectious diseases [25,91].

Drought intensifies these nutritional stresses by drastically reducing forage biomass, increasing plant fiber content, and worsening deficiencies in phosphorus, copper, zinc, selenium, and cobalt [29]. Long-distance migration in search of grazing areas further strains camels’ metabolism. Water scarcity slows foregut motility and fermentation, heightening risks of impaction, gas accumulation, and atony of the first stomach compartment (C1) [53].

### 5.4. Feeding of Neonatal and Growing Camel Calves

Proper nutrition in the early life of the camel calves is essential since growth, immunity, and long-term productivity of the camel require appropriate colostrum feeding, milk feeding, and early rumen (C1/C2) functional maturation. Camel calves are relatively slow-growing, have extended maternal milk reliance, and are slow to intake solid feed, differences that should be incorporated in customized feeding plans as opposed to other domesticated ruminants. The consumption of colostrum in the initial 2–4 h of birth is necessary. Passive transfer failure predisposes calves to diarrhea, septicemia, and retarded growth. Due to the variation in quality of colostrum according to parity, season, and maternal nutrition, it is essential to monitor the nutrition of dams towards the end of gestation in order to attain sufficient immunity in the neonates [88,96,97]. Excessively fibrous feeds, though, may hamper rumen compliance and motility to cause more risk of indigestion or impaction [12,33,68,88].

Weaning in pastoral systems is usually at 4–6 months, although in vigorously controlled dairies, this may be postponed to aid constant growth [72,98,99]. Appropriate neonatal nutrition is a significant factor in improving survival, health, and later productivity [96]. Growth performance of growing camels under various feeding regimes has been quantified. Studies show average daily gains ranging from 174 to 565 g/day for male camels under different protein levels and up to 810–840 g/day in well-fed Sudanese calves provided 12–14% crude protein diets. Daily dry matter intake was approximately 1.44% of body weight in camels vs. 2.5% in cattle calves, illustrating species-specific intake patterns relative to energy and protein requirements [100].

### 5.5. Feeding of Pregnant and Lactating Camels

The most nutritionally active physiological periods among dromedary camels are pregnancy and lactation. These are times that demand well-calculated rations that will be conducive to fetal growth, maternal health, quality of colostrum, and sustained milk production [97]. Therefore, to prevent metabolic disorders, reproductive failure, and the lack of calf performance, specific feeding in gestation and lactation should be made [9,83,101]. It is observed that the lactating camels, which produce 5–10 L of milk on a daily basis, demand about 0.9–1.1 kg of crude protein per day and around 65–80 MJ ME per day, which are variable based on their milk production and environmental factors but are still lower than dairy cows yielding comparable volumes of milk when adjusted for body weight [68,102]. These comparatively low requirements are the result of camels’ longer foregut retention time, high urea recycling efficiency, and lower endogenous nitrogen losses [49,50,55].

#### 5.5.1. Nutrition During Pregnancy

The gestation period in a camel takes between 13 months, and in this time, the nutrient requirement increases at a slow pace, but it is in the last three months that the third trimester proves to be the most crucial one [88,103,104,105]. Lack of energy results in over-mobilization of hump fats, high levels of ketone bodies, and a low body state that may affect the growth of placental development and fetuses.

The late gestation protein needs are very high to supply the fetal tissues, colostrum, and mammary growth. Poor nutritional amounts of crude protein (low <5–6% in dry-season forage) may lead to failure of passive transfer in neonatal calves since immunoglobulin levels are damaged. Trace minerals, especially phosphorus, copper, cobalt, zinc, and selenium, are required for the placental functions, antioxidant defenses, and development of the fetal immune system [30,33,106,107]. The failures are observed in the form of undeveloped calves, prolonged placenta, lowered fertility, and high periparturient morbidity [12,108,109]. It is also important to have access to clean water on a regular basis. Despite the ability of camels to endure dehydration, a long-term deprivation of water during pregnancy causes a decrease in blood volume and viscosity and impairs uteroplacental perfusion [96].

#### 5.5.2. Feeding During Lactation

The uniqueness of the nutrient requirements during lactation is due to the fact that camels are relatively high in water, vitamin C, and bioactive compounds in their milk. With management, nutrition, and breed, milk yield is maintained up to 8–18 months. Protein and energy consumption should be raised significantly, preventing too much depletion of the hump, chronic loss of weight, and decreased milk production [32,68,69,110].

The amount of minerals required during lactation is a lot higher. Phosphorus is necessary to produce milk and to generate energy, whereas selenium, copper, and zinc play a role in immunity and oxidative balance. Extensive dairy systems that depend on irrigated fodder are especially susceptible to the lack of Se, Cu, Zn, and Co, which lowers the yield of milk and increases the levels of oxidative stress indicators [68,88]. During pregnancy and lactation, feeding has increased efficiency in reproduction, calf survival, maternal health, and subsequent productivity [9,66,83,101]. Apparent dry matter digestibility (DMD) in dromedary camels has been reported at approximately 57.5–76.7%, depending on forage type, while crude protein digestibility (CPD) improves with higher dietary protein and digestible energy levels (*p* < 0.05) in growing camels [46].

### 5.6. Feeding of Breeding Males

The male camels have a distinct seasonal variation in food consumption, hormonal state, and exercise, which affects nutritional requirements and clinical susceptibility as compared to females [111]. In the rut, testosterone levels are elevated very high, leading to aggression, irritability, frequent marking behavior, and decreased feed consumption. Most males also gain much body condition at this time because of increased physical activity, accompanied by a reduction in appetite [90]. To avoid excessive fat depletion and weight loss, it is important that the diet is of sufficient energy density. Quality forage, including alfalfa, with a moderate level of concentrates, can help balance the energy without predisposing to C1 dysmotility and acidosis states, to which camels are especially susceptible in case of excessively high levels of the concentrates [7,36,112].

Protein consumption should be adequate to facilitate spermatogenesis, accessory gland activity, and muscle support. Low levels of crude protein in the diet—as in dry-season desert forages—may reduce semen volume, sperm motility, and total fertility. The trace minerals include zinc, selenium, copper, and cobalt, which are necessary for testicular functions, protection against oxidants, and the production of hormones. The mineral deficiencies that are well reported in irrigated fodders and desert pastures [91,111]. Heat stress, dehydration, and large amounts of salt in the diet, especially in grazing systems that engage a lot of halophytes, may adversely impact the quality of semen and electrolytes. Male breeding should thus be observed to have unrestrained access to clean water, ample shade, and regulated salt content [113].

## 6. Nutritional Pathologies and Metabolic Disorders

Nutritional imbalances in camels are characterized by exceptionally complicated systemic interactions. GIT-related disorders are often spread along the gut–liver–kidney axis, affecting the hepatic metabolism, renal clearance, electrolyte balance, and systemic inflammatory reactions [7]. Recent research based on the combination of ultrasonography, hemato-biochemistry, oxidative stress biomarkers, and cytokine profiling has progressed the diagnosis and knowledge of nutritional diseases such as Johne's disease, hepatic fibrosis [72,114], renal abscessation [35], oxidative stress caused by trypanosomiasis [77,80]. Various disorders have been identified in camels that feed on mineral-contaminated diets, inappropriately designed concentrates, or anthropogenic waste, including urolithiasis, gastric impaction, obstruction of the esophagus, or pica [115,116]. These circumstances indicate the necessity of species-specific nutrition quality standards and new diagnostic systems in the near future.

### 6.1. Energy and Carbohydrate Imbalances: Acidosis, Ketosis, and Negative Energy Balance

Diagnostic imaging has now become a revolution in camel nutritional pathology. Diagnosis of impaction, obstruction, peritonitis, hepatic steatosis, renal insufficiency, and systemic effects of malnutrition are now in the focus of ultrasonography of the abdomen, liver, kidney, and gastrointestinal compartments [53,56,114].

The slow C1 motility of the camel, its conservative fermentation, and restricted buffering secretion are by themselves predisposing factors to upsets in foregut pH when rapidly fermentable carbohydrates are abruptly presented [6,79]. Subacute and severe gastric (C1) acidosis develops when fermentation of starch in the diet exceeds the salivary buffering and absorption of C1 by the camel. When the concentration of food intake is high, lactate-fermenting bacteria increase, creating a drop in C1 pH and repressing regular fibrolytic microbes [53,56]. As with cattle, camels experience osmotic fluid changes to C1, decreased motility, and death of microbes, but the clinical expression is usually more insidious. Wang et al. [40] observed that C1 stagnation is worsened by dehydration, thereby making a person more susceptible to the condition of acid-base imbalance. Affected camels have intermittent anorexia, low rumination, mild colic, and hard and scanty feces. The chronic cases develop into chronic dyspepsia, which is a fluctuating appetite and decreased milk yield syndrome that has been widely reported in the literature related to the gastroenterology of camels [12,76,117]. The animals can be found in serious cases as weak, severely dehydrated, tachycardic, recumbent, or even dead under the influence of endotoxemia.

These disturbances are further increased by environmental stressors. A typical hindrance of a desert environment is water restriction, which decreases C1 motility and raises the osmolarity, enhancing acidosis [73,84]. Heat stress decreases food consumption and changes energy allocation to thermoregulation, which is a factor in NEB. According to Tharwat and El-Deeb [59], heat-stressed camels show high indicators of oxidative stress, which indicates that environmental stress and metabolic impairment are interrelated. Forage losses caused by drought lead to a decrease in the availability of both energy and protein to the point where animals are forced to enter chronic fat mobilization and be predisposed to subclinical ketosis, particularly where lactation needs are high.

Camels that are fed irrigated forages or fed concentrated feeds in the absence of sufficient fiber tend to develop SARA-like disorders because of decreased chewing activity and a deficiency of saliva buffering. Smuts and Bezuidenhout [40] noted that the anatomy of the camel stomach, in general, the lack of an omasum, leads to differing flow characteristics than cattle, which means they are unable to stabilize large changes in dietary fermentability. Consequently, the foregut ecosystem also turns unstable due to current feeding habits, which exposes them to metabolic instability [40,72,118]. It involves prevention by ensuring rigid observance of camel-related feeding principles. These encompass slow introduction of gradual concentrates, roughage sustenance, high-starch supplement avoidance, mineral equilibrium (especially P, Co, and Cu), and perpetual water availability. It is imperative that body condition, milk yield, feeding behavior, and C1 motility be closely monitored to diagnose the disease at an early stage. Finally, the incompatibility between the evolutionary physiology of camels and the demands of the present-day feeding conditions highlights the importance of species-specific nutritional thresholds and evidence-based feeding solutions [30,31].

#### Protein and Nitrogen Metabolic Disorders of Dromedary Camels

Protein and nitrogen disorders in dromedary camels arise primarily from a mismatch between highly efficient nitrogen-conservation mechanisms and modern feeding practices. Excessively degradable protein sources, inadequate fermentable energy, dehydration, and impaired C1 motility collectively disrupt nitrogen capture, increasing the risk of ammonia accumulation despite otherwise low dietary protein levels [9]. Nevertheless, given the overconsumption of fast-degrading protein by animals, such as irrigated forages or lush green fodders, or dietary protein meals rich in protein, the frequency of ammonia being emitted exceeds the assimilation ability of the microbes [62]. The presence of high soluble protein in the diet with insufficient fermentable energy contributes to the fact that the nitrogen is not captured well, which leads to the absorption of ammonia. Dietary energy is of particular concern in times when hump lipolysis supplants carbohydrate metabolism, during the late dry season and early lactations, when camels are particularly susceptible [64,80].

Clinically, ammonia toxicity in camels often develops insidiously, with subtle behavioral and production changes preceding overt neurological signs, reflecting the species’ slow foregut kinetics and delayed systemic absorption. The illness is similar to bovine ammonia toxicity, except that camels will exhibit slower development of the condition, as the motility of the foregut is slower. According to Guerouali et al. [62], camel dyspepsia was reported in a number of cases, whereby high levels of C1 ammonia contributed to producing systemic neurological symptoms. The dehydration that slows C1 dilution also increases ammonia accumulation, and this finding is also corroborated by the dehydration physiology of Schmidt-Nielsen and Knut [26]. Reduced C1 motility (usually challenged by heat stress, mineral deficiencies, or low-fiber diets) decelerates outflow and promotes further uptake of ammonia into the systemic circulation. Lack of microbial activity results in high blood urea nitrogen (BUN), ineffective C1 fermentation, loss of appetite, and loss of weight. It has especially been observed in camels that are being fed irrigated forages with low concentrations of essential trace minerals [9,82,119], since microbial inefficiency causes the liver to continually rid the body of ammonia, placing significant metabolic strain.

The malfunctioning of the urea cycle is further weakened by liver dysfunction, which can be brought about by plant toxins, chronic malnutrition, or systemic inflammatory disease. According to Tharwat and El Deeb [59], metabolically stressed camels demonstrate increased oxidative stress indicators and a change in liver enzymes, which means that liver insufficiency may be associated with nutritional stress. In cases where hepatic detoxification is ineffective, the ammonia level becomes high in the blood, resulting in the manifestation of encephalopathy-like symptoms [80]. Furthermore, C1 dysmotility, which often goes hand-in-hand with low-quality roughage, dehydration, and overconsumption of highly fermentable sources, such as Tharwat and Al-Sobayil [6], is an impediment in the recycled nitrogen distribution, which decreases the availability of this nutrient to microbial syntheses.

Even protein deficiency is a cause of nitrogen disorders. Desert forages in drought situations usually have less than 4–5% crude protein, which is not enough to sustain protein production by the microbes. Lack of sufficient nitrogen causes microbial fermentation to go very slowly, leading to a decrease in the production of VFA and inhibition of energy metabolism. The resultant anorexia and state worsen the total nitrogen intake further, forming a vicious cycle of malnutrition [62,120,121]. Smuts and Bezuidenhout [39] stressed that the anatomy of a camel's stomach, particularly the sacculated C1 compartments, relies on the integrity of the microbes and that the lack of nitrogen has a direct adverse effect on the stability of fermentation. Adult camels have been shown to maintain nitrogen balance at crude protein intakes approximately 20–30% lower than those required for cattle at maintenance, largely due to enhanced urea recycling and reduced urinary nitrogen losses [7,53,112].

### 6.2. Minerals and Vitamins in Dromedary Camels: Functions, Requirements, and Clinical Implications

The trace minerals and vitamins are also vital to the camel’s immunity, bone metabolism, antioxidant defense, and reproductive functions. Nevertheless, significant local shortages of selenium, copper, cobalt, zinc, iodine, and vitamins A and E have been reported mainly in dry soils that have been drained by climatic change, overgrazing, and low dietary variety [12,57,122,123]. Specifically, selenium deficiency has far-reaching effects because the selenoproteins, including glutathione peroxidase (GPx), play a central role in protecting against oxidative damage [124]. Even though most studies involving selenium and its effects are done in cattle and sheep, recent translational studies indicate that similar oxidative stress, apoptotic, and mitochondrial imbalance could occur in camel tissues in case of mineral deficiency [125,126]. Similarly, deficiency of copper and molybdenum (which is usually due to high concentrations of copper) has been linked to anemia, impaired immunity, poor fertility, coat changes, and exposure to infectious disease [17,119]. With the increase in modern camel production, nutritional attention is given to close monitoring of the interactions, bioavailability, and supplementation approaches of the mineral.

One of the most common mineral disorders in camels is phosphorus deficiency. Forage phosphorus concentrations in arid and semi-arid rangelands frequently fall below 0.12–0.18% dry matter, compared with estimated camel requirements of approximately 0.25–0.30% dry matter for maintenance and lactation, explaining the widespread occurrence of pica and osteodystrophy [74,89,120,127]. Forages grown in the desert, and especially in the dry season, have very low levels of phosphorus, which causes animals to mobilize skeletal reserves over extended intervals. The clinical manifestations are pica, bone pain, stiffness, poor growth, low fertility, weak neonatal calves, and osteomalacia [11,126]. Pica behavior, which appears in consuming bones, stones, soils, or even plastic, is widely reported in pastoral areas of Pakistan, Sudan, and the Sahel and has a great correlation with soils that are phosphorus-depleted [95,128]. Since phosphorus is vital in the production of ATP and fermentation by microbes, its deficiency causes inadequate energy metabolism, low milk production, and chronic loss of weight. The laboratory results usually involve hypophosphatemia, low serum alkaline phosphatase, and low total protein as a result of decreased microbial production.

Copper deficiency is not an exception because in irrigated fodder systems, molybdenum, sulfur, and iron antagonize the absorption of copper [122,129,130]. Camels with copper deficiency appear depigmented in the coat, with weak hair, anemic, poor immunity, lose their fertility, and suffer from bone fragility. Tharwat and El-Deeb [59] reported high oxidative stress levels (MDA) with low antioxidant enzyme activity (SOD, GPx) in camels in a deficient state of trace minerals under copper conditions and a metabolic stress condition. Young animals exhibit retarded growth, and breeding males can exhibit reduced libido or have abnormal sperm morphology. The laboratory manifestations are low serum copper, low ceruloplasmin, and microcytic anemia [11,86].

Poor wound healing, decreased immunity, reproductive failure, and skin disorders are some of the effects of zinc deficiency. The result of zinc-poor diets (which are typical of cereal-based feeding systems and of certain irrigated forage systems) is parakeratosis, alopecia, low-quality hooves, and retarded puberty. Due to the need for zinc in preserving the integrity of the epithelium and immune enzyme activity, deficiencies expose camels to chronic dermatological and respiratory infections [25,91,131]. In the case of marginal deficiencies, serum zinc levels can be normal, and thus clinical evaluation is necessary.

Deficiency of selenium, which usually coexists with vitamin E deficiency, affects antioxidant defenses, muscular performance, and reproductive performance; see Table 2. The inadequate levels of selenium in soils, which are prevalent in arid and irrigated lands, lead to low forage selenium and later myodegeneration, weak calves, retained placenta, and a higher incidence of systemic infections [125,132,133]. Deficiency of selenium is closely linked with a high level of glutathione peroxidase deficiency in blood and an increase in the oxidative stress biomarkers. Neonatal weakness, acute collapses, or respiratory failure can be confused with infectious diseases, and therefore, diagnosis may be difficult without the use of biochemical tests [58,134].

**Table 2:** Volatile fatty acid production patterns and energy metabolism characteristics in dromedary camels.

Mineral	Estimated Requirement *	Field Deficiency Status in Camel Regions	Significant Clinical/Nutritional Consequences	References
Phosphorus (P)	3–4 g/day (adults); 6–8 g/day (late gestation/lactation)	Very common in desert and sandy soils	Pica, osteodystrophy, poor reproductive performance, reduced C1 motility	[76,128]
Copper (Cu)	10–12 mg/day	Widespread; exacerbated by high molybdenum soils	Anemia, poor coat quality, immune suppression, and infertility	[55,119,135,136,137]
Cobalt (Co)	0.1–0.2 mg/kg DM	Typical due to low Co soils	Reduced microbial synthesis, anorexia, weight loss, chronic dyspepsia	[122,138,139,140]
Zinc (Zn)	40–60 mg/day	Moderate in desert systems	Skin lesions, poor wound healing, reduced fertility	[25,91,137,141]
Selenium (Se)	0.1–0.3 mg/day	Patchy; low in sandy rangelands	Oxidative stress, myopathy, weak neonates	[72,122,125,133,142]
Calcium (Ca)	3–6 g/day	Less common; Ca:P imbalance more relevant	Bone disorders, reduced milk yield	[17,76]
Sodium (Na)	5–10 g/day	Standard in halophyte-only diets (excess)	Salt toxicity, dehydration, foregut stasis	[17]

* Estimated requirements based on available camel studies and extrapolated ruminant nutrition guidelines, reflecting approximate daily needs under maintenance conditions; actual requirements may vary with physiological and environmental factors. The table presents reported ranges of volatile fatty acid proportions, fermentation characteristics, and associated metabolic features, emphasizing camel-specific differences in energy utilization compared with cattle.

Affected camels consistently show significantly elevated malondialdehyde concentrations and reduced activities of antioxidant enzymes (GPx, SOD), often declining by 20–40% compared with clinically healthy animals under similar management conditions [16]. The absence of iron deficiencies in adult camels is because of grazing on desert soils rich in iron, whereas young calves kept on milk-only diets do not have iron. Parasites are also a chronic cause of blood loss. Affected calves have microcytic anemia, pallor, retarded growth, and low hematocrit [16,17,86].

Although calcium deficiency is less commonly reported in camels than in cattle, it arises at the end of gestation and early lactation period when food Ca: P ratios are disproportionate. The deficiency of calcium nutrients leads to weak bones, poor milk production, and subclinical hypocalcemia, particularly in instances where the phosphorus-deficient diets disrupt the homeostasis of the minerals [76].

These mineral disorders are complicated by vitamin deficiencies. Deficiency of vitamin A is observed in the course of long dry seasons, which leads to the keratinization of the mucous membranes, night blindness, fertility failure, and predisposition to respiratory infections; see Table 2. The intake of carotene in the diet is strongly connected to the level of vitamin A, and it drastically reduces in regions characterized by drought. The camels use liver stores, and hence, deficiency occurs gradually but is extreme at an advanced stage [18,85].

**Figure 2:**
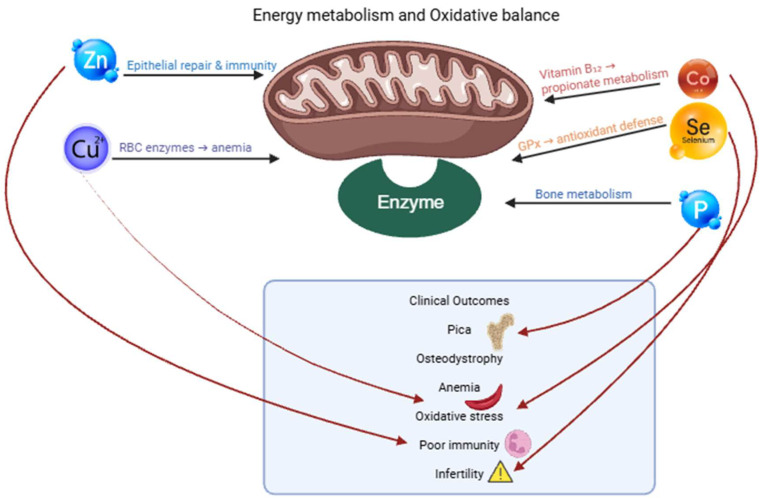
Mineral–metabolic interaction network illustrating nutrition-related systemic disturbances in dromedary camels. This figure depicts the interrelationships among mineral imbalances, metabolic dysregulation, and organ-level effects in camels, highlighting how deficiencies and toxicities affect hepatic, renal, and digestive functions through interconnected biochemical pathways.

The deficiency of vitamin E plays a very important role in oxidative stress, muscular degeneration, impaired immunity, and poor reproductive results, as depicted in Figure 2. Tharwat and El-Deeb [59] emphasized that vitamin E and selenium are frequently found to be at a lower level in metabolically stressed camels, which is a result of their combined action as antioxidants. The clinical signs are muscle weakness, stiff gait, low level of milk production, and high morbidity of the calf [32,71].

### 6.3. Oxidative Stress–Related Disorders

The other area that is currently evolving at a high pace is the place of nutrition in camel immunity and oxidative physiology. The immunological peculiarities of camels, such as heavy-chain-only antibodies and specific cytokine reaction patterns, are also essential factors influenced by nutrition. Malondialdehyde (MDA), enzymes like superoxide dismutase (SOD), catalase, GPx, and total antioxidant capacity are some of the oxidative stress biomarkers that have been widely investigated as markers of metabolic stress, inflammation, infectious disease, and dietary imbalance [59,143]. These biomarkers are particularly applicable in high-performance camels (such as racing and dairy camels subjected to heat load, long-distance transport, or high-energy fed diets) [113]. Oxidative stress assessment inclusion in dietary examination provides a complex, evidence-based model of early identification of subclinical deficiencies, metabolic imbalances, and over-nutrition.

Once the acidosis is severe, the integrity of the epithelium is worse, which allows the translocation of endotoxins and leads to systemic inflammation. Tharwat et al. [59] showed the higher amount of oxidative stress biomarkers, such as MDA, and decreased activity of SOD/GPx in camels exposed to metabolic stress, suggesting that acidosis is one cause of systemic oxidative damage. Heat stress exacerbates dehydration, electrolyte imbalances, and oxidative stress, as evidenced by elevated malondialdehyde levels and altered antioxidant enzyme activities such as glutathione peroxidase and superoxide dismutase [69,80,144].

### 6.4. Gastrointestinal Disorders Associated with Nutritional Imbalance

The most common gastrointestinal complication with nutritional stress, particularly in lengthy drought periods, is impaction. The camels in dry seasons feed on the highly lignified, low-moisture shrubs, thorny Acacia species, coarse crop residues, and halophytes, most of which have very low protein and are very difficult to digest [17,95]. Dehydration, which occurs in hot months and on long walks, also retards C1 motility. Hansen and Schmidt-Nielsen [38] showed that water depletion significantly decelerates the rate of digesta and elevates the level of viscosity within the foregut, which predisposes the camels to stagnation. Consequently, ingesta builds up and forms solid masses that block C1 and C2 compartments. The camels become anorectic, have progressive abdominal distention, dehydration, meager dry feces, and ruminate less. According to Tharwat and Al-Sobayil [6], chronic dyspepsia and C1 atony are characteristic syndromes of prolonged feeding on poor-quality roughage and insufficiency in terms of hydration.

Foreign-body impaction is also another problem in peri-urban or garbage-covered settings. Camels that browse in the markets, rubbish areas, or even on the roads swallow plastic bags, ropes, clothes, cardboard, and other things that cannot be digested. These accumulate in C1 and give rise to trichobezoars, or huge conglomerates, which in most cases necessitate surgical management [15,116,145]. Tharwat [53,56,114] underlines that the importance of such impactions is that they seem to mimic nutritional impaction but can be more severe, which is usually related to severe dehydration, electrolyte imbalance, and secondary endotoxemia.

Another big gastrointestinal manifestation is pica, which is closely associated with mineral deficiencies, specifically phosphorus, copper, cobalt, and, in some cases, sodium. The effects of chronic phosphorus-deficient diets that are prevalent in desert areas and irrigated fodder technology include ingestion of bones, stones, soil, wood, and, in some cases, plastic by camels [67,146]. Pica is not a behavioral interest but is a direct physiological reaction to mineral deprivation, and it plays a large role in the ingestion of foreign objects that culminate in impaction; see Table 3. Suttle et al. [17] point out that phosphorus deficiency is among the strongest stimuli of pica in grazing herbivores, which is consistently supported in camel herds around the Sahel, Arabian Peninsula, and South Asia. The deficiency of cobalt also lowers appetite and distorts the rumen microbial synthesis, predisposing animals to abnormal ingestion behavior.

### 6.5. Diagnostic Imaging and Ultrasonography of Nutritional Disorders

Since nutritional diseases in camels do not always lead to local effects but rather result in complex and multisystemic effects, including the gastrointestinal tract, liver, and kidneys, imaging will give the necessary insight into pathophysiological alterations, which cannot be identified solely by clinical examination [35,74,112]. The causes of nutritional imbalances are usually located in the gastrointestinal tract, particularly in the sacculated chambers C1 and C2, but the imbalances spread by metabolic routes and immunological processes to affect hepatic functions, renal integrity, and systemic homeostasis; see Table 3.

Hepatic involvement is rapid and occurs as a result of decreased nutrient absorption and microbial activity. There is an overloading of the liver with the by-products of modified fermentation—ammonia, bacterial toxins, lactate, and oxidative metabolites. Tharwat and El-Deeb [59] showed that camels who are subjected to nutritional stress have high liver enzymes, oxidative stress-related products (MDA), and low antioxidant enzyme activity (SOD and GPx), which means that hepatic oxidative metabolism is seriously impaired. Hepatic changes are also caused by chronic copper deficiency, which is common in irrigated fodder systems [119], since low copper concentrations cause ceruloplasmin production to decrease and oxidative protection to be compromised. These hepatic imaging results are in line with biochemical aberrations of high AST, GGT, low albumin levels, and bilirubin changes; see Table 3.

**Figure 3:**
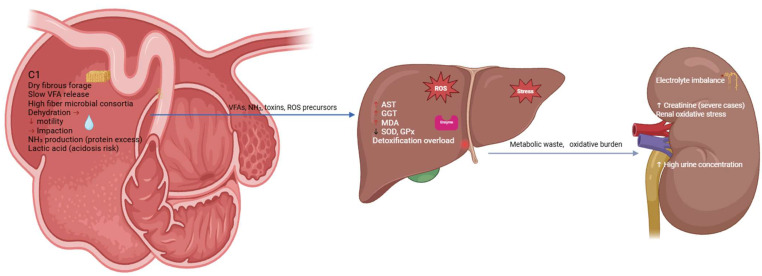
Integrated gut–liver–kidney axis illustrating the progression of nutrition-related metabolic disorders in dromedary camels. The schematic demonstrates how primary nutritional disturbances originating in the foregut can propagate through hepatic metabolism and renal function, contributing to systemic pathology. The figure emphasizes the multi-organ nature of camel nutritional disorders and their diagnostic complexity. ↑ represents an increase in value, ↓ represents a decrease or decline, and → represents smooth flow or leads to.

Metabolic disturbances tend to progress to the kidney to establish a metabolic axis of gut–liver–kidney. The nutritional deficiencies, especially phosphorus, zinc, and selenium, change the performance of renal perfusion, glomerular filtration, and tubular metabolism, as shown in Figure 3. Dehydration, as in pastoral systems, and aggravated by drought, is concentrated on nephrotoxic metabolites and inhibits renal functioning [83,114]. Renal changes such as higher cortical echogenicity, loss of corticomedullary differentiation, and poor renal size in the chronic cases are seen ultrasonographically. Acid toxicity or ammonia toxicity (due to hampered microbial fermentation or excessive soluble protein consumption) [61,62] also puts strain on the kidney by elevating acid excretion requirements. Tharwat [56] observed that metabolic syndrome, as in camels, is often characterized by hepatic and renal lesions occurring simultaneously, which explains how nutritional pathology affects the entire system.

The combination of clinical, biochemical, and imaging results offers the greatest accuracy of diagnosis. As an example, a camel with anorexia, pica, less rumination, and dullness can be a symptom of phosphorus deficiency, impaction, and copper imbalance. These are distinguished with the aid of ultrasonography as foregut impaction, hepatocellular echogenic changes are identified, or renal compromise. The diagnosis is supported by laboratory tests that reveal hypophosphatemia, low serum copper, increased liver enzymes, and a change in the signs of oxidative stress [59]. This combined method enables the detection of secondary metabolic effects before they turn into irreparable damages to the organs.

### 6.6. Disorders Occurring During Pasture Feeding: Toxic Plants and Forage-Related Poisoning

Toxic plant consumption in pasture grazing is a large yet poorly diagnosed cause of nutritional pathology among dromedary camels. Despite the fact that camels are well known to be selective browsers and capable of eating thorny, saline, and fibrous plants with relative safety [24], camels are still susceptible to a broad range of the toxic principles of desert shrubs, halophytes, as well as opportunistic weeds—particularly during periods of drought, overgrazing, or seasonal unavailability of food; see Table 4. In this case, the camels will be pushed to feed on the poor or foreign vegetation, making them more prone to intoxication. Indiscriminate foraging and pica are other factors that are related to nutrient deficiency, especially phosphorus, copper, and cobalt, which further predispose the camels to toxic ingestion of plants [48,117]. The African and Arabian Peninsulas are covered with halophytes in most of the camel grazing areas. Although the plants are important as a source of forage products subjected to drought, their high sodium, oxalate, and sulfate contents present metabolic hazards [47,150]. Sodium overload causes dehydration and worsens the already existing water scarcity, as Hansen and Schmidt-Nielsen [38] indicated that camels with a salt load have reduced foregut motility and enhanced osmotic stress. Concurrently, shrubs with oxalates in them absorb calcium in the gastrointestinal tract, causing hypocalcemia, weakness, and, in severe cases, nephrotoxicity as a result of the calcium oxalate precipitation. Similar neurological effects are also caused by sulfate toxicity, which destabilizes thiamine metabolism and induces camels to polioencephalomalacia-type syndromes, especially at periods when there is low availability of dietary fiber and thiamine [151,152].

Toxic ingestion of plants has a very unpredictable clinical manifestation, which varies according to toxin dosages, plant species, camel nutritional state, and stressors in the environment; see Table 4. Symptoms common to them are anorexia, drooling, abdominal pain, diarrhea or constipation, rumen stasis, weakness, photosensitivity, jaundice, and neurological dysfunction, including staggering or seizures. Due to the toxicity of vegetation, secondary metabolic changes (accumulation of ammonia, deficiency in detoxification, hypoglycemia, and oxidative imbalance) are common consequences [121,153]. In cases of pyrrolizidine alkaloid toxicity, ultrasonography can identify hepatomegaly, hepatic echogenicity increases, or biliary obstruction. Plant toxicity is highly augmented by drought and overgrazing. Where limited palatable forage is available, camels turn to otherwise undesirable species, such as those containing high levels of terpenes, nitrates, and oxalates. Mineral deficiencies contribute to this forced browsing; for example, phosphorus-deprived camels have pica and feed on woody shrubs but unintentionally ingest poisonous plant foods [9]. Furthermore, the rapid intake stimulated by hunger doubles the dose of toxins per unit of time and overloads the hepatic detoxification systems. The liver of the camel is resilient in its ability to break down plant secondary compounds, whereas excessive exposure or malnutrition that follows simultaneously undermines this adaptive benefit.

**Table 4:** Scientific characterization of camel feed types with digestibility, anti-nutritional factors, fermentation kinetics, and nutritional risk.

Feed Type	Digestibility (%)	Fiber (NDF/ADF)	Anti-Nutritional Factors	Fermentation Pattern	Nutritional Risks	Key References
Halophytes	42–55%	Very high	Oxalates, salt	Slow, stable VFA	Salt toxicity; dehydration	[4,31,71,81]
Acacia Browse	45–60%	High	Tannins	Moderate fermentation	Protein binding → deficiency	[4,81]
Desert Grasses	50–65%	High	Silica	Moderate VFA	Impaction during drought	[14,154]
Crop Residues (straw)	40–50%	High lignin	Silica, lignification	Slow fermentation	Chronic dyspepsia	[1,14,117]
Green Fodder (irrigated)	60–75%	Moderate	Low minerals	Fast fermentation	Acidosis if abrupt	[117]
Restaurant Waste	65–90%	Low	Mycotoxins; starch overload	Rapid lactate production	Acidosis, liver toxicosis	[15,113]
Garbage Feeding	—	—	Plastics, toxins	—	Foreign body syndrome	[15,113,116,145]

### 6.7. Disorders Caused by Feeding on Garbage or Human Food Waste

The leftovers of restaurants, particularly spoiled carbohydrates like fermented rice, stale bread, pasta that has been thrown out, and cooked grains, are an added risk to acute and subacute lactic acidosis. The buffering secretion and the more conservative fermentation ecosystem of camels are particularly sensitive to the sudden increase in the intake of rapidly fermentable carbohydrates, compared to cattle [33,68,71]. Consumption of large amounts of old cooked rice or sweetened bakery waste by camels results in rapid overgrowth of lactate-producing bacteria in C1, with the resultant effects of a sharp drop in pH, foregut atony, abdominal pain, dehydration, systemic acidosis, and, in severe cases, circulatory collapse. The presentation is almost identical to subacute and acute ruminal acidosis in other ruminants, except that camels tend to develop it more slowly and develop a chronic dyspepsia-type progression [46,62].

From a metabolic point of view, garbage-related feeding disorders cause a series of metabolic hiccups: electrolyte dysregulation, acidosis, liver failure, kidney failure, cardiovascular failure, and oxidative stress. Diagnosis is based on the combination of clinical manifestations (anorexia, bloating, dullness), biochemical findings (acidosis, increased AST/GGT, hypophosphatemia), and ultrasonographic (impression or hepatic alterations) findings. Chronic foreign body accumulation often needs surgical intervention [15,149].

## 7. New Knowledge and Studies in the Camel Feeding

### 7.1. Feed Evaluation Techniques

In vitro digestibility techniques enable the researcher to develop an estimate of the disappearance of dry matter (DM) and organic matter (OM) in the presence of camel-specific inoculum based on C1 contents. Due to the existence of a special fermentation microbiota in the camel foregut that has been adapted to the arid-zone vegetation, camel digestibility values are frequently undervalued when using conventional cattle inoculum [154]. Experiments involving camel inoculum reflect better digestibility of tannin-containing Acacia species and salt-tolerant shrubs as a result of the adaptation of the microbes to these types of food. Reduced digestibility of lignified desert shrubs is associated with field effects of weight loss and dyspepsia during seasonality [46,105].

The gas production methods are used as a supplement to the digestibility tests in order to evaluate the kinetics of fermentation, rate, lag time, and total amount of the gas. Production of gas indicates the presence of microbial activity and the length of degradability of the substrate [155]. The complexity of the fiber components in desert halophytes can be associated with slow yet maintained fermentation curves, whereas carbohydrate-rich irrigated forages have fast fermentation curves, which in the field may predispose camels to subacute acidosis [120,127]. Gas profiles have been useful in coming up with safer concentrate supplementation programs for dairy camels.

Near-infrared spectroscopy (NIRS) has emerged as a useful tool in the quick estimation of forage quality, that is, fiber fractions, crude protein, moisture, minerals, and phenolic compounds. NIRS has been used to map the seasonal variation in browse quality of camel forages in the Arabian Peninsula and East Africa. Due to the extremely variable nature of camel rangelands and the frequently remote areas, NIRS is used to monitor pastoralists in a non-destructive and rapid way. Copper, phosphorus, and zinc deficiencies are usually associated with metabolic disorders, and NIRS can correlate the mineral analyses with those in laboratories [156,157]. Microbial profiling has also shown that camel dehydration, mineral imbalance, or dietary manipulation causes significant changes in C1 microbial communities, which impairs fermentation and predisposes camels to atony, impaction, and metabolic instability [59,121]. These results demonstrate the importance of uniformity in the diet and mineral balance in maintaining the health of microbes.

### 7.2. Feed Additives and Supplements

Feed supplements are considered to have a larger role in camel nutritional optimization, microbial recovery, increased fiber digestibility, and the prevention of metabolic disorders. Though the majority of the additives used are borrowed from cattle nutrition, special responses in camels are under clear explanation [46,120,127].

The enzymes, like cellulases, hemicelluloses, and xylanases, enhance the breakdown of fiber, particularly in halophytes and desert shrubs that contain a high lignin content. Enzyme supplementation applies in the period of drought or when animals feed on low-quality browse. Better digestion of fiber minimizes the risk of impaction, and C1 motility is reduced in dehydrated camels [61]. Buffer compounds such as sodium bicarbonate, magnesium oxide, and other alkali compounds are becoming a necessity in the camel dairies, where concentrate feeding is being applied. Buffers also improve stability in rumen pH and inhibit subacute acidosis, prevalent when camels are fed on large quantities of carbohydrate-rich diets [34,158]. Perennial low-grade acidosis affects protein synthesis by microbes and adds to liver stress, which confirms the importance of buffer supplementation in high-yielding dairy herds.

Vitamin and mineral blends can hardly be done without in the current camel diets, particularly since most desert forages are deficient in phosphorus, copper, cobalt, zinc, selenium, and vitamins A and E [90]. Such premixes inhibit pica, metabolic bone disease, oxidative stress, anemia, reproductive inefficiency, and immunosuppression. The supplementation of vitamin E and selenium is related to antioxidant defense, which is consistent with the previous studies by Tharwat and El-Deeb [59] who found oxidative stress-related biomarkers in nutritionally stressed camels.

### 7.3. Molecular and Omics Approaches

Nutrigenomics studies the gene expression of camels under the influence of nutrients. Early research shows that the state of energy and minerals and inflammatory changes the hepatic and metabolic gene networks. The insight into such shifts helps to develop diets that regulate the resilience of metabolism. Metabolomics, such as metabolite profiling on blood and C1 fluids, gives a clue to the real-time metabolic responses to food [89,105]. The metabolomic signatures assist in the detection of the initial signs of acidosis, ketosis-related conditions, mineral malnutrition, or oxidative stress. They complement the use of biochemical profiling and can be useful in the detection of early disease, especially in high-producing camels [64,104].

The characterization of the camel foregut compartments by microbiomes has indicated a wide range of fibrolytic bacteria and archaea, protozoa, and fungi with a unique adaptation to salty, tannin-containing, and lignified forage. Alterations in the population of microbes are linked to nutritional conditions such as impaction, acidosis, and dyspepsia [46,120]. Microbiome dynamics can be used to improve the design of probiotics, optimize the use of dietary fiber, or prevent dysbiosis when changing feed.

### 7.4. Quality of Feeding and Sustainable Nutrition

Accurate feeding and sustainable nutrition are becoming necessary as camel production changes to commercial dairy and meat systems and the rangelands experience pressure on the environment. Precision in feeding is associated with the monitoring of the intake of nutrients, real-time adjustment of rations, and the reduction in waste and maintenance of productivity. Development of camel-specific camel ration formulation models remains scarce. Existing feeding guidelines depend a lot on cattle information, which opens loopholes in the specifications of the camel, especially in minerals, vitamins, and energy metabolism. The filling of these gaps is one of the main priorities in the future, as numerous gaps are reported by Suttle et al. and Lopez and Miranda [17,119].

The nutrition of the camel on its sustainable rangelands is still the focus of the camel ecology. Overgrazing, shrub depletion, and desertification cause a decrease in the nutritive plants and an increase in the rate of toxic plant ingestion [92]. Such practices as controlled grazing, rotation, salt-tolerant forage farming, and planting of drought-resistant shrubs can be classified as sustainable practices. These methods favor long-term forage resilience and decrease the incidence of nutritional infirmities [29]. To conclude, precision feeding and sustainable rangeland management are the future of camel feeding, which combines technology, ecology, and physiology in order to keep the productivity high without endangering animal welfare and environmental stability.

## 8. Future and Prospects and Future Research Requirements

One of the key research gaps is the lack of standardized, camel-specific feeding guidelines. The majority of existing recommendations are only extrapolated on the basis of cattle or small ruminant values, although there are known differences in the anatomy of camel digestion, water metabolism, nitrogen recycling, and microbial ecology [38,40,159]. These extrapolations are dangerous to undervalue mineral needs, inadequately calculate energy requirements, and overlook camel peculiarities, including sensitivity to fast-fermentable carbohydrates, slow buffering rates, and unique recycling of nitrogen [82]. The urgent requirement of the modern camel dairies is the nutrient requirement table calculated on the basis of empirical research using C1 fermentation kinetics, metabolizable energy system, and trace mineral metabolism peculiar to camels. According to Sadan et al. and Aljumaah et al. [15,68], mineral deficiencies are common in the herds of camels as a result of the use of low-quality desert forages, but there is a paucity of systematic studies determining optimal levels of different minerals to maintain, grow, lactate, get pregnant, and work camels [68,116].

The other important area of focus is creating omics-based precision nutrition models. Promises in transcriptomics, metabolomics, metagenomics, and proteomics have already started to unveil the hidden association of diet, health, and productivity in a more profound, meticulous way [103,118]. Tharwat and El-Deeb [59] presented the evidence of the oxidative stress reactions and metabolic disruptions in the case of nutritional disorders and the necessity to combine the information on the molecular level with the dietary control. The next round of research needs to integrate nutrient intake data with gene expression alterations, metabolic pathway modifications, and microbiome dynamics to model adaptive feeding patterns, which could predict nutritional stress prior to clinical manifestations. This type of omics-informed precision nutrition would lower the rates of impaction, acidosis, syndromes of mineral deficiency, and syndromes of hepatic-renal metabolic disorders.

Future studies should also investigate the interconnection between nutrition and disease resilience, especially immune performance, fertility, and stamina. Nutritional stress, particularly the lack of copper, selenium, zinc, vitamin E, and energy, has far-reaching impacts on oxidative balance, immunological cell performance, and reproductive hormones [21,22,24]. These deficiencies in camels increase their vulnerability to infectious diseases, negatively affect the conception rate, and condition animals to a systemic metabolic failure [59]. A combination of nutritional biomarkers and the study of the relations between reproductive physiology and epidemiology of diseases would help understand how balanced diets increase resilience in heat-stressed and drought-prone environments.

Besides this, there exist novel prospects provided by the advent of genomics, nutrigenomics, and microbiome science. The genome sequencing of camels has helped to demystify the genetic diversity, breed composition, and the mechanism of adaptation [43,44]. Tests in microbiomes show that the camelids have very specialized fibrolytic and halophilic microbial communities that help them to feed on saline desert shrubs and lignified forages. The knowledge of such systems will enable the development of microbiome-specific nutritional interventions, probiotics, and feed additives based on camel physiology.

## 9. Limitations in Camel Feeding

Although there has been an increasing scientific interest in camel nutrition, significant constraints impede the achievement of proper feeding management in the traditional pastoral and the contemporary semi-intensive systems [11,83,86]. As these limitations are due to scientific knowledge gaps, ecological, socioeconomic, and camel physiological complexities. Collectively, these problems undermine nutritional sufficiency, productivity, and disease resistance. One of the major constraints is that there are no developed, species-specific feeding criteria for dromedary camels [43,160]. The majority of nutrient requirements applied in the area are still based on the extrapolation of cattle and small ruminants, even though the differences in the digestive anatomy, nitrogen recycling, water metabolism, and feed selectivity of camel rumens are well documented [9,82].

The other constraint is the poor nutritional value of available forages in major camel-rearing areas. The desert rangelands, which are also typified by halophytes, thorny shrubs, and drought-resistant grasses, are usually low in crude protein, high in lignin, over-supplemented in salts, and unbalanced in minerals [95]. One of the weaknesses that is related to management is the inconsistent mineral supplementation. In spite of the prevalent deficiencies of phosphorus, copper, cobalt, zinc, selenium, and vitamins A and E [17,119], regular or non-routine supplementation is used. Due to feeding limitations, there are abnormal and unsafe feeding behaviors in the peri-urban system where camels often feed on garbage, plastic, domestic waste, or left-out cooked food items, particularly old rice and bread. The materials lead to foreign-body impaction, microbial contamination, mycotoxicosis, lactic acidosis, and hepatic or renal injury [53,54,114]. The move towards urban camel rearing has superseded the invention of appropriate feeding standards, and producers in this area are left to rely on inexpensive yet harmful waste products.

Ecological constraints such as degradation of rangelands, biodiversity of native forage, and infestation by invasive toxic plants are long-term challenges. Climate pattern changes and nutritional stability in camel ecosystems are becoming more unstable, which increases the requirement to stabilize feeding and forage restoration approaches to camels [105,111,160]. Altogether, camel feeding restrictions can be attributed to the combination of physiological peculiarities, gaps in science, environmental stress, and socioeconomic limitations. To deal with them, a combination of research, feeding standards specific to camels, better mineral supplementation plans, sustainable rangeland operations, and the implementation of precision nutrition systems is needed.

## 10. Conclusions

During this review, it is clear that nutritional disorders in camels can hardly stay within the gastrointestinal system. Although the sacculated C1 and C2 compartments provide an impressive adaptability in the digestion of coarse, lignified, and salty forage, these compartments are [105,111,160] highly susceptible to sudden dietary shifts, water deprivation, and mineral imbalance [7,35]. The first pathway to more complicated systemic manifestations is dysmotility, impaction, subacute acidosis, and dysbiosis. Nutrient deficiencies, especially of phosphorus, copper, cobalt, zinc, selenium, and vitamins A and E, are still prevalent in the camel-rearing areas and are a key factor contributing to pica, poor microbial fermentation, immunosuppression, oxidative stress, and infertility [71,105].

The complex gut–liver–kidney metabolic axis in the camels is demonstrated by the metabolic disorders, including hepatic insufficiency, ketosis-like energy deficits, ammonia toxicity, and oxidative stress syndromes. Tharwat and El-Deeb [59] have managed to reveal the fact that camels are experiencing nutritional stress, which leads to major changes in hepatic enzymes, lipid peroxidation, and antioxidant defenses impairment—highlighting the systemic effects of a long-term nutritional imbalance. On the same note, the interrelation between the nutritional dyspepsia, C1 stasis, and metabolic deterioration was also reported by Tharwat and Al-Sobayil [6,78,79], and they confirmed that the early gastrointestinal dysfunction is an early sentinel event of more severe metabolic compromise.

Recent innovations in the method of feed evaluation, such as in vitro digestibility tests, gas generation fermenters, and the NIRS-based forage evaluation, have helped in getting a better interpretation of the camel feed resources. Nutrigenomics, metabolomics, and microbiome profiling are molecular measures that are starting to demonstrate the dietary influence on gene activity, fermentation pathways, and metabolic stress responses [161]. These instruments are promising in the development of omics-based precision nutrition models, which eventually can anticipate nutritional imbalance in the preclinical stage of disease development [103,162]. However, there are still great constraints. The feeding standards of camels are not sufficiently developed, and most of the recommendations are based on data that are extrapolated by taking into account the cattle that have well-established physiological differences [31,106]. Considering these issues, this review highlights the critical role of interdisciplinary research (bringing together nutrition, physiology, microbiology, pathology, ecology, and precision technology). Calibration of entire camel-specific nutrient requirement programs, mapping of regional resources of feeds, verification of omics-based nutritional biomarkers, and encouraging sustainable rangeland management are the key steps in enhancing camel health and productivity. In both traditional and peri-urban systems, education among the community and veterinary assistance will also help alleviate the feeding-related disorders.

## Figures and Tables

**Table 3:** Diagnostic–biochemical framework for major nutritional disorders in dromedary camels integrating ultrasonographic findings, biochemical biomarkers, and underlying pathophysiological mechanisms.

Disorder	Primary Nutritional Etiology	Ultrasonographic Findings	Biochemical Indicators	Pathophysiological Mechanism	Key References
C1 Impaction	Dry fibrous forage; dehydration	Immobile ingesta; reduced contractions	↑PCV, ↑TP, mild alkalosis	Dehydration → motility ↓ → ingesta stasis	[39,147,148]
Subacute Acidosis	Restaurant waste (rice, bread), sudden concentrate	Fluid ingesta; mucosal edema	↓pH, ↑lactate	Rapid fermentation → lactic surge	[15,116,145]
Chronic Dyspepsia	Low minerals, poor digestibility	Very low C1 motility	Mild ↑AST/GGT; hypophosphatemia	Microbial collapse → reduced fermentation	[6,95,147]
Ammonia Toxicity	Excess soluble protein; NPN misuse	Gas distension; mild fluidity	↑NH3; respiratory alkalosis	Excess microbial proteolysis → hepatic overload	[9,64,80,149]
Hepatic Lipidosis/Toxicosis	Moldy feed; plant toxins	Hyperechoic liver; capsular tension	↑AST, ↑GGT, ↑MDA	Toxin → hepatocellular degeneration	[74,75]
Mineral Deficiency Complex	P–Cu–Zn–Se-deficient forage	Non-specific	↓Trace minerals; anemia	Impaired enzymatic/metabolic pathways	[17,122]

This table synthesizes key nutrition-related disorders affecting dromedary camels, linking primary dietary etiologies with characteristic ultrasonographic features, associated biochemical alterations, and the principal mechanisms driving disease development. The framework emphasizes the interaction between feeding practices, dehydration, and camel-specific digestive physiology in the manifestation of both subclinical and clinical metabolic disorders. C1, first stomach compartment; PCV, packed cell volume; TP, total protein; pH, hydrogen ion concentration; AST, aspartate aminotransferase; GGT, gamma-glutamyl transferase; NH_3_, ammonia; NPN, non-protein nitrogen; MDA, malondialdehyde; P, phosphorus; Cu, copper; Zn, zinc; Se, selenium. ↑represents an increase in value, ↓represents a decrease or decline, and →represents smooth flow or leads to.

## Data Availability

The original contributions presented in this study are included in the article. Further inquiries can be directed to the corresponding author.
